# Supplementary material for: Impact of an Extremely Dry Period on Tree Defoliation and Tree Mortality in Serbia
Source: Plants (Basel). 2022 May 11;11(10):1286. doi: 10.3390/plants11101286 (PMC9144404; doi:10.3390/plants11101286)
Supplement: Supplementary file 1 [file plants-11-01286-s001.zip › plants-1627661-supplementary.pdf]

Supplementary Material

Article: **Impact of an Extremely Dry Period on Tree Defoliation and Tree Mortality in Serbia**

Authors: Goran Češljar**,** Filip Jovanović, Ljiljana Brašanac-Bosanac, Ilija Đorđević, Suzana Mitrović, Saša Eremija, Tatjana Ćirković-Mitrović and Aleksandar Lučić

This PDF file includes:
Tables S1 to S9
Figures S1 to S5

Table S1. Trends in defoliation on trees during the years of research with the final outcome of dying and division into groups

SP Level I	Locality	Altitude (m)	Stand age **	Tree Species	Defoliation in percentages (%) per year															Group
					2004	2005	2006	2007	2008	2009	2010	2011	2012	2013	2014	2015	2016	2017	2018	
429	Smederavska Palanka	114	61-80	Ulmus minor	*										5	0	0	0	100	II
424	Andrvlje Testera	225	61-80	Quercus petraea	35	35	35	25	10	15	25	20	35	30	25	15	10	10	100	II
421	Vršački Breg	370	61-80	Quercus petraea	10	15	20	20	15	30	35	30	30	20	30	25	30	30	100	I
415	Maljen	630	61-80	Fagus sylvatica	10	30	10	10	0	10	5	0	0	10	0	40	75	80	100	I
406	Jamnjaci	1400	81-100	Picea abies	30	20	5	0	10	0	0	0	0	0	25	30	10	10	100	II
404	Bunatovac	1120	41-60	Fagus sylvatica	0	0	0	10	0	20	10	10	5	0	0	0	0	0	100	II
404	Bunatovac	1120	41-60	Fagus sylvatica	10	5	0	10	0	0	40	15	10	0	10	0	10	30	100	I
404	Bunatovac	1120	41-60	Fagus sylvatica	0	0	0	10	0	10	20	10	0	5	10	10	10	0	100	II
99	Vranjska Banja	868	41-60	Fagus sylvatica	0	5	0	25	15	15	10	0	0	5	0	15	30	40	100	I
96	Muhovac	850	41-60	Castanea sativa	*										10	10	0	10	100	II
60	Vrnjačka Banja	392	41-60	Quercus frainetto	25	20	15	20	0	10	15	0	10	10	50	60	60	60	100	I
420	Gobeljska reka	1558	81-100	Picea abies	10	0	0	5	5	0	5	0	0	0	0	0	0	100		II
420	Gobeljska reka	1558	81-100	Abies alba	10	0	0	10	10	5	5	0	0	5	5	5	5	100		II
101	Deliblato	125	81-100	Robinia pseudoacaccia	75	75	75	75	60	55	50	50	30	35	75	70	80	100		III
73	Pobijenik	1201	uneven aged	Picea abies	15	10	5	10	15	15	5	0	5	10	20	0	25	100		II
42	Despotovac	386	uneven aged	Acer campestre	*									10	30	40	95	100		I
28	Potaj Čuka	619	61-80	Fagus sylvatica	0	0	0	0	0	0	0	0	0	0	0	0	0	100		II
24	Oreškovica	189	<= 20	Robinia pseudoacaccia	0	10	0	40	10	30	0	10	10	0	50	30	95	100		I
21	Grošnica	591	uneven aged	Fagus sylvatica	*							0	15	90	85	70	60	100		I
430	Vranić	165	21-40	Ulmus minor	*										10	85	100			I
427	Kupinske Grede	70	101-120	Fraxinus angustifolia spp. Oxycarpa	15	25	25	30	20	25	45	40	35	35	75	85	100			I
425	Raškovica	75	81-100	Fraxinus angustifolia spp. Oxycarpa	65	15	10	45	20	15	30	35	25	50	10	40	100			I
418	Murtenica	1344	81-100	Abies alba	*							15	0	0	0	0	100			II
418	Murtenica	1344	81-100	Abies alba	*							0	0	0	0	0	100			II
403	Pekare	915	61-80	Fagus sylvatica	0	0	0	10	0	10	0	5	15	40	40	70	100			I
106	Popovica	425	101-120	Tilia platyphyllos	5	10	0	0	0	10	20	10	5	0	0	0	100			II
106	Popovica	425	101-120	Quercus petraea	15	15	15	15	15	20	35	20	25	15	15	15	100			II
106	Popovica	425	101-120	Fagus sylvatica	5	5	0	5	5	10	5	5	5	0	0	0	100			II
69	Bela Palanka	1355	81-100	Fagus sylvatica	30	30	30	40	70	80	70	90	60	0	10	0	100			III
67	Makrešane	268	21-40	Quercus frainetto	10	10	10	10	20	10	20	30	20	30	90	90	100			I
56	Lazac	383	61-80	Quercus frainetto	35	35	35	35	30	10	10	15	0	10	80	80	100			I
21	Grošnica	591	uneven aged	Populus tremula	0	15	10	5	0	5	5	5	30	10	25	60	100			I
415	Maljen	630	61-80	Abies alba	0	10	0	10	10	20	0	10	10	40	80	100				I
415	Maljen	630	61-80	Abies alba	0	10	10	0	10	0	5	0	0	20	60	100				I
415	Maljen	630	61-80	Abies alba	0	10	0	10	20	10	10	10	0	30	70	100				I
402	Tara II	1151	81-100	Picea abies	30	40	10	5	10	10	0	0	0	0	5	100				II
402	Tara II	1151	81-100	Picea abies	40	50	20	10	25	20	5	0	0	20	25	100				II
402	Tara II	1151	81-100	Picea abies	50	55	20	10	10	10	0	0	0	15	30	100				I
402	Tara II	1151	81-100	Picea abies	5	10	5	5	10	0	0	0	0	0	0	100				II
94	Poganovo	616	41-60	Quercus cerris	0	0	0	40	35	40	40	20	10	90	90	100				I
50	Brezova	860	81-100	Fagus sylvatica	5	5	0	5	30	20	10	0	0	15	80	100				I
33	Bukova Glava	432	121-140	Quercus petraea	10	40	10	30	10	20	10	10	10	10	10	100				II
23	Turija	339	41-60	Carpinus betulus	0	0	0	0	10	0	30	0	30	50	90	100				I
15	Struganik	406	21-40	Quercus cerris	20	20	10	0	10	10	10	0	0	10	40	100				I
15	Struganik	406	21-40	Quercus cerris	20	20	0	10	10	0	10	0	0	20	20	100				II
15	Struganik	406	21-40	Quercus cerris	30	20	10	10	20	10	10	0	0	20	30	100				II
14	Cer - Sever	70	41-60	Carpinus betulus	0	20	10	20	5	10	0	0	0	10	90	100				I
7	Valjevo	268	41-60	Quercus frainetto	30	20	10	20	10	10	20	10	20	10	95	100				I
23	Turija	339	41-60	Acer campestre	10	20	20	10	20	30	30	10	10	70	100					I
20	Tometino Polje	632	61-80	Betula pendula	20	20	10	10	10	5	5	20	20	80	100					I

SP Level I	Locality	Altitude (m)	Stand age **	Tree Species	Defoliation in percentages (%) per year															Group
					2004	2005	2006	2007	2008	2009	2010	2011	2012	2013	2014	2015	2016	2017	2018	
20	Tometino Polje	632	61-80	Betula pendula	20	25	15	15	10	0	0	5	60	85	100					I
14	Cer - Sever	70	41-60	Carpinus betulus	0	10	10	10	10	10	5	10	10	40	100					I
28	Potaj Čuka	619	61-80	Fagus sylvatica	10	0	0	20	15	10	5	10	5	0	100					II
56	Lazac	383	61-80	Fagus sylvatica	25	25	25	25	15	10	10	10	25	95	100					I
69	Bela Palanka	1355	81-100	Fagus sylvatica	20	0	10	5	0	0	0	10	5	5	100					II
415	Maljen	630	61-80	Fagus sylvatica	30	30	10	10	0	10	10	5	0	10	100					II
415	Maljen	630	61-80	Fagus sylvatica	20	20	20	10	10	10	5	0	0	80	100					I
20	Tometino Polje	632	61-80	Populus tremula	30	35	30	15	10	10	10	15	20	40	100					I
20	Tometino Polje	632	61-80	Prunus avium	15	20	20	20	15	5	30	30	90	95	100					I
15	Struganik	406	21-40	Quercus cerris	20	30	10	10	10	10	0	5	0	10	100					II
19	Stragari	251	61-80	Quercus cerris	15	10	15	15	5	0	5	0	5	5	100					II
19	Stragari	251	61-80	Quercus cerris	15	10	15	15	0	0	10	5	5	0	100					II
19	Stragari	251	61-80	Quercus cerris	20	20	20	15	0	0	0	5	10	0	100					II
19	Stragari	251	61-80	Quercus cerris	15	15	15	20	5	0	0	0	5	10	100					II
19	Stragari	251	61-80	Quercus cerris	15	20	20	10	0	0	10	5	10	15	100					II
29	Rudna Glava	346	21-40	Quercus cerris	10	20	20	20	0	0	10	10	70	95	100					I
29	Rudna Glava	346	21-40	Quercus cerris	20	50	10	20	80	90	50	70	80	80	100					III
35	Jabukovac	136	41-60	Quercus cerris	0	30	10	10	0	20	30	15	25	0	100					II
36	Kladovo	168	41-60	Quercus cerris	20	40	10	20	10	0	10	20	90	95	100					I
81	Kuršumlija	453	21-40	Quercus cerris	20	20	10	30	10	20	20	10	30	80	100					I
7	Valjevo	268	41-60	Quercus frainetto	40	20	10	10	10	0	20	20	10	95	100					I
26	Ranovac	216	61-80	Quercus frainetto	*		10	10	30	30	10	40	50	80	100					I
36	Kladovo	168	41-60	Quercus frainetto	10	20	20	20	0	0	20	5	20	80	100					I
37	Vratarnica	231	41-60	Quercus frainetto	20	40	40	40	30	0	10	0	0	70	100					I
37	Vratarnica	231	41-60	Quercus frainetto	10	10	40	20	10	0	10	0	10	0	100					II
41	Rekovac	400	41-60	Quercus frainetto	40	40	20	30	20	20	30	30	40	15	100					I
41	Rekovac	400	41-60	Quercus frainetto	*		0	50	60	60	50	60	95	40	100					III
56	Lazac	383	61-80	Quercus frainetto	30	25	20	20	5	0	10	5	0	10	100					II
103	Odžaci	75	<= 20	Quercus robur	10	5	15	15	30	35	35	30	25	95	100					III
426	Grabovačko Vitonajevačko ostrvo	0	121-140	Quercus robur	50	55	35	20	25	35	35	75	80	80	100					III
24	Oreškovica	189	<= 20	Robinia pseudoacaccia	10	0	10	10	0	0	0	0	20	40	100					I
41	Rekovac	400	41-60	Robinia pseudoacaccia	20	20	15	40	70	80	80	65	60	95	100					III
68	Brus	328	61-80	Robinia pseudoacaccia	0	0	0	90	40	40	0	60	80	90	100					III
401	Tara I	1098	101-120	Abies alba	20	25	20	10	5	5	0	5	10	0	100					II
415	Maljen	630	61-80	Abies alba	20	10	0	20	20	10	10	30	80	90	100					I
415	Maljen	630	61-80	Abies alba	20	10	10	10	20	0	0	5	0	0	100					II
417	Zlatar	1354	61-80	Picea abies	5	5	0	0	5	5	10	5	5	75	100					I
59	Kraljeva kamenica	572	<= 20	Pinus sylvestris	15	20	15	10	0	0	0	0	15	15	100					II
59	Kraljeva kamenica	572	<= 20	Pinus sylvestris	5	5	5	5	0	0	0	0	10	10	100					II
59	Kraljeva kamenica	572	<= 20	Pinus sylvestris	15	15	10	5	0	0	10	0	10	10	100					II
21	Grošnica	591	uneven aged	Betula pendula	10	10	10	5	30	30	0	0	70	100						I
21	Grošnica	591	uneven aged	Betula pendula	15	15	15	10	0	10	0	0	90	100						I
13	Povlen	1035	61-80	Fagus sylvatica	20	30	10	20	15	10	0	0	0	100						II
21	Grošnica	591	uneven aged	Fagus sylvatica	10	5	0	0	0	0	0	0	10	100						II
50	Brezova	860	81-100	Fagus sylvatica	35	30	20	20	30	0	0	0	0	100						II
51	Srednja Reka	1263	81-100	Fagus sylvatica	*						10	80	90	100						I
403	Pekare	915	61-80	Fagus sylvatica	0	0	0	0	0	0	10	20	85	100						I
29	Rudna Glava	346	21-40	Quercus cerris	10	40	10	10	20	20	10	10	30	100						I
100	Golemo Selo	634	121-140	Quercus cerris	10	10	10	40	40	40	30	10	40	100						III
30	Kučevo	217	61-80	Robinia pseudoacaccia	10	30	30	80	40	50	70	90	90	100						III
415	Maljen	630	61-80	Abies alba	0	20	10	20	30	10	0	80	80	100						I
101	Deliblato	125	81-100	Pinus nigra	45	70	60	60	80	85	80	80	80	100						III

SP Level I	Locality	Altitude (m)	Stand age **	Tree Species	Defoliation in percentages (%) per year															Group
					2004	2005	2006	2007	2008	2009	2010	2011	2012	2013	2014	2015	2016	2017	2018	
40	Svetozarevo	421	41-60	Fagus sylvatica	0	0	0	0	10	10	5	0	0	100						II
96	Muhovac	850	41-60	Fagus sylvatica	0	0	0	0	70	90	40	30	30	100						III
409	Mali Jastrebac	569	81-100	Fagus sylvatica	10	10	10	10	5	10	5	15	10	100						II
102	Plavna	75	<= 20	Populus hybrides	40	40	10	40	20	35	40	25	35	100						III
102	Plavna	75	<= 20	Populus hybrides	*							65	60	100						I
96	Muhovac	850	41-60	Fagus sylvatica	*				20	25	0	0	100							II
21	Grošnica	591	uneven aged	Betula pendula	20	15	20	15	15	20	10	0	100							II
23	Turija	339	41-60	Fraxinus ornus	10	20	20	10	20	95	20	30	100							I
60	Vrnjačka Banja	392	41-60	Quercus cerris	15	20	10	15	0	10	10	20	100							II
17	Srezojevci	554	41-60	Quercus frainetto	25	15	15	40	40	70	5	0	100							II
23	Turija	339	41-60	Carpinus betulus	10	20	10	10	30	80	90	90	100							III
51	Srednja Reka	1263	81-100	Fagus sylvatica	*						10	10	100							II
95	Topli Do	1230	41-60	Fagus sylvatica	20	0	0	80	90	90	90	100								III
64	Mozgovo	685	61-80	Quercus petraea	0	0	10	20	20	20	70	100								I
96	Muhovac	850	41-60	Castanea sativa	0	0	5	20	20	30	0	100								II
40	Svetozarevo	421	41-60	Fagus sylvatica	0	0	0	0	90	90	80	100								I
48	Požega	455	61-80	Fagus sylvatica	35	30	5	15	0	50	50	100								I
421	Vršački Breg	370	61-80	Fraxinus ornus	0	0	0	10	10	25	100									II
75	Prijepolje	1050	41-60	Quercus petraea	60	70	30	30	40	45	100									III
20	Tometino Polje	632	61-80	Betula pendula	15	20	5	10	25	15	100									II
102	Plavna	75	<= 20	Populus hybrides	35	30	40	30	25	15	100									II
102	Plavna	75	<= 20	Populus hybrides	15	25	10	35	15	10	100									II
18	Spomen Park KG	256	81-100	Quercus frainetto	25	35	30	30	20	15	100									II
27	Osanica	652	41-60	Quercus petraea	20	20	20	40	35	35	100									I
105	Čortanovačka šuma	175	61-80	Tilia platyphyllosc	15	10	10	20	10	5	100									II
23	Turija	339	41-60	Carpinus betulus	60	50	50	40	90	100										III
416	Petkovica	214	101-120	Quercus frainetto	40	60	50	30	20	100										
102	Plavna	75	<= 20	Populus hybrides	80	80	80	80	70	100										III
412	Tisovac	1145	41-60	Fagus sylvatica	10	0	20	10	0	100										II
426	Grabovačko Vitonajevačko ostrvo	0	121-140	Quercus robur	65	65	65	80	100											III
427	Kupinske Grede	70	101-120	Quercus robur	40	45	45	90	100											III
37	Vratarnica	231	41-60	Quercus frainetto	10	40	50	70	100											III
427	Kupinske Grede	70	101-120	Quercus robur	50	55	55	65	100											III
106	Popovica	425	101-120	Quercus petraea	20	20	15	100												II
427	Kupinske Grede	70	101-120	Quercus robur	65	70	90	100												III
418	Murtenica	1344	81-100	Abies alba	10	10	20	100												II
427	Kupinske Grede	70	101-120	Quercus robur	65	75	100													III
21	Grošnica	591	uneven aged	Quercus cerris	35	40	100													III
418	Murtenica	1344	81-100	Abies alba	20	20	100													II
73	Pobijenik	1201	uneven aged	Abies alba	25	25	100													I
48	Požega	455	61-80	Fagus sylvatica	5	5	100													II
423	Kolut Kozara	70	121-140	Quercus robur	90	90	100													III
105	Čortanovačka šuma	175	61-80	Tilia platyphyllosc	65	100														III
41	Rekovac	400	41-60	Robinia pseudoacaccia	60	100														III

* A new tree has been singled out at this site and its monitoring has begun.

** Stand age according to ICP Forest methodology (see: https://icp-forests.org/documentation/Dictionaries/d_tree_age.html)

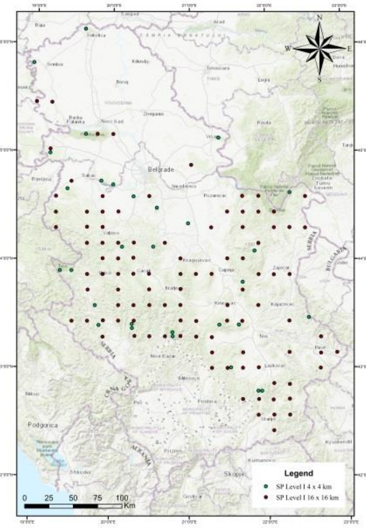

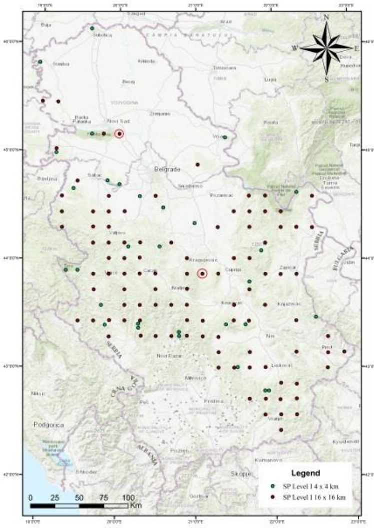

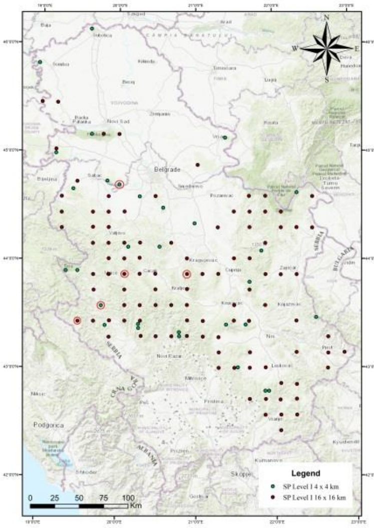

2004	2005	2006

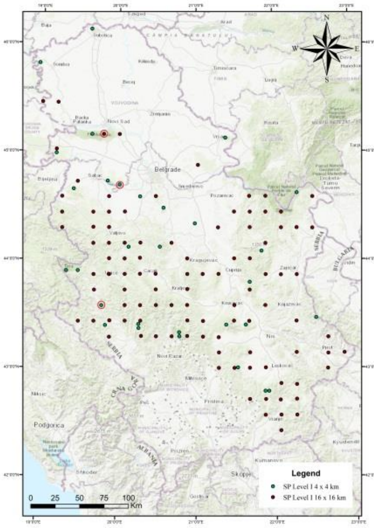

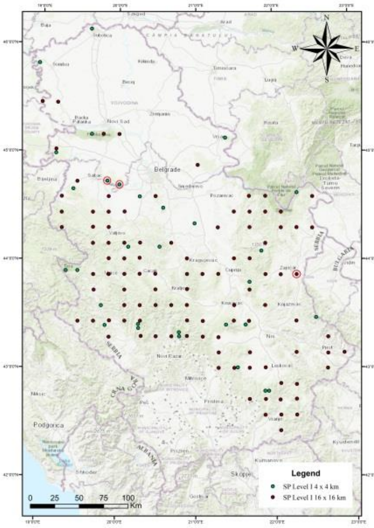

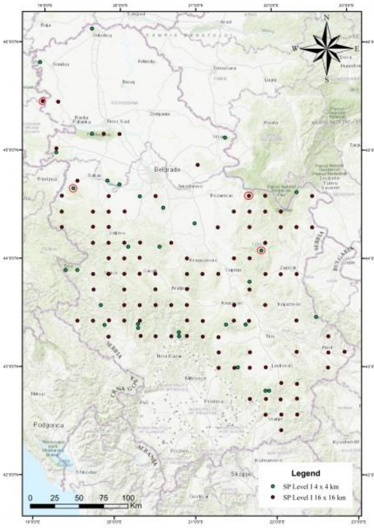

2007	2008	2009

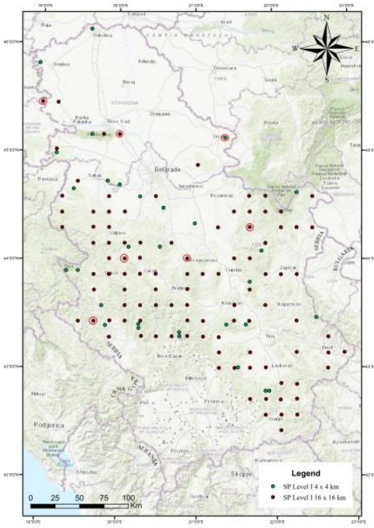

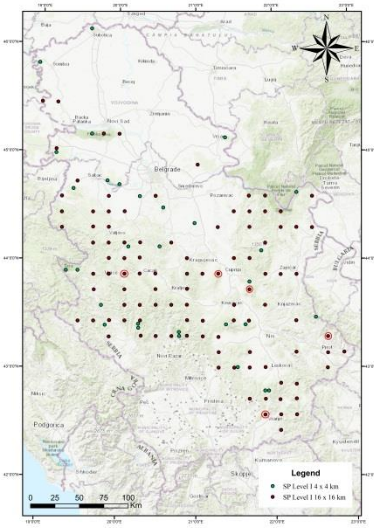

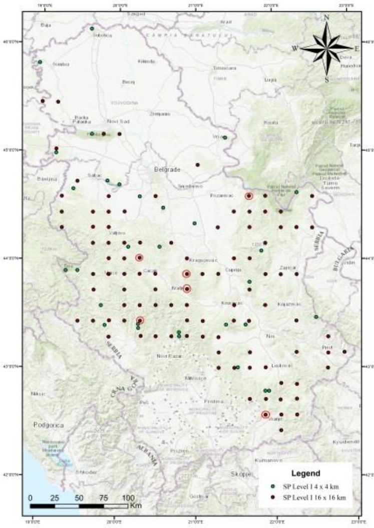

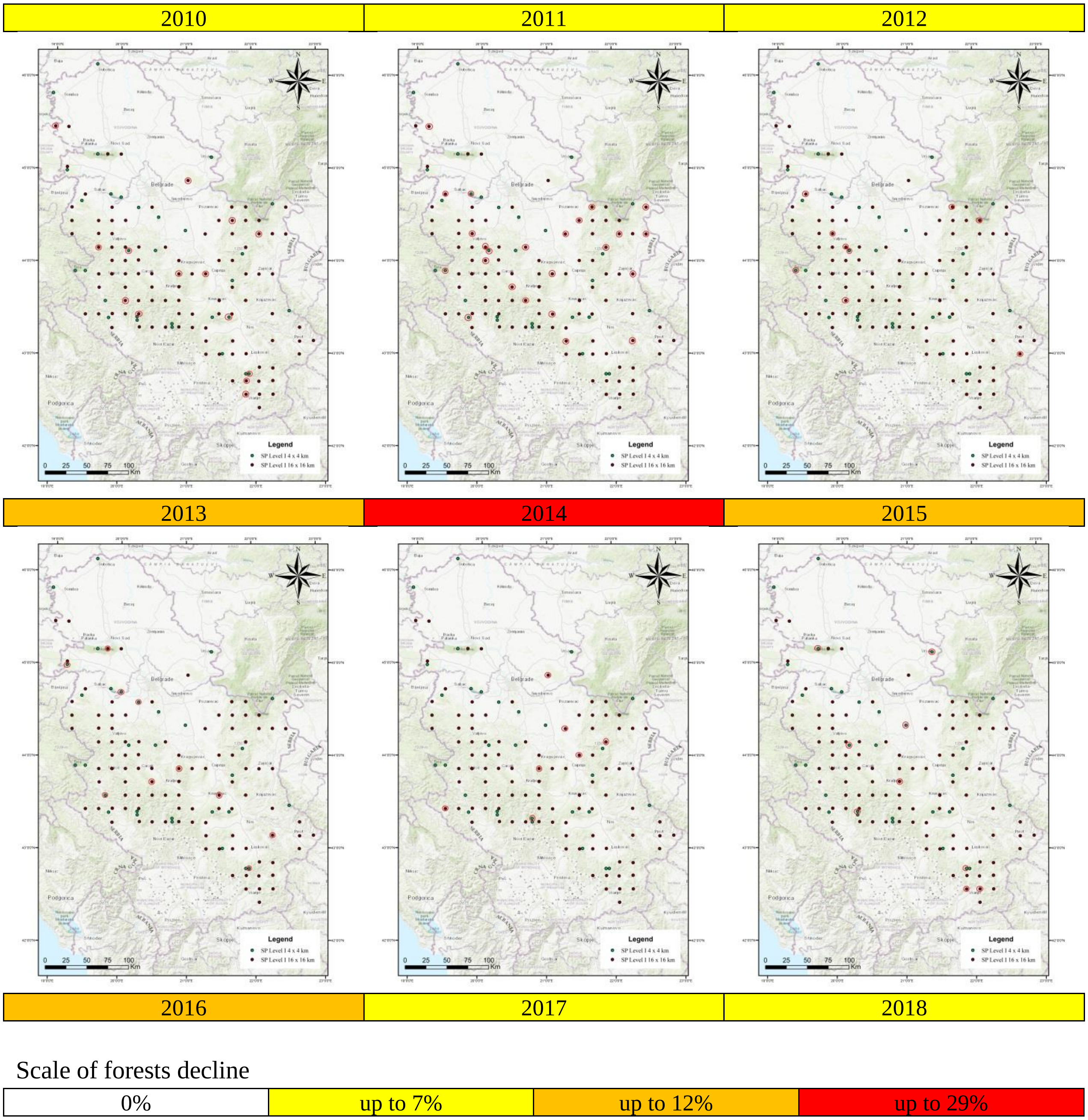

Figure S1. Forest decline on sample plots on the territory of the Republic of Serbia in the period from 2004 to 2018. ʘ - Sample plots where tree mortality or groups of trees has been recorded (defoliation 100%).

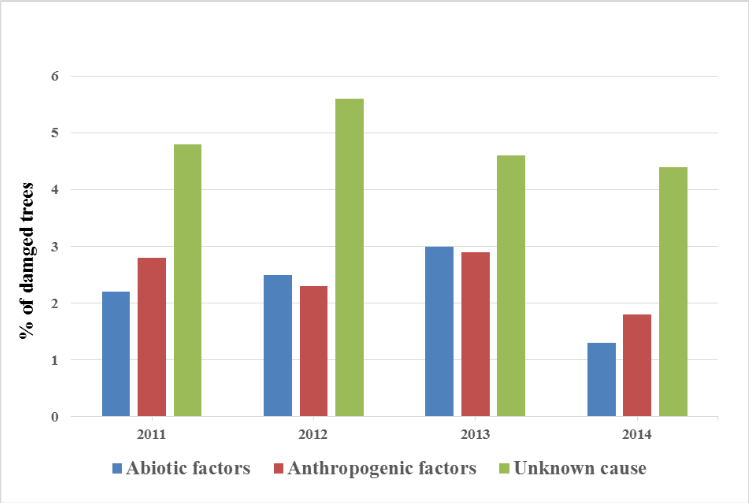

Figure S2. The percentage of trees of all species with the damage on the sample plots (2011-2014)

Table S2. Descriptive and nonparametric statistics for the annual mortality rates of broadleaf and conifer tree species monitored in the territory of Serbia in the period of 2014–2018.

Tree type	Sample size	M	MAD	MIN	MAX	Average rank in KWt	Test statistic	*P*-value
Broadleaves	29	0.003	0.003	0.000	0.078	16.828	1.0037	0.3164
Conifers	5	0.009	0.009	0.000	0.492	21.400		

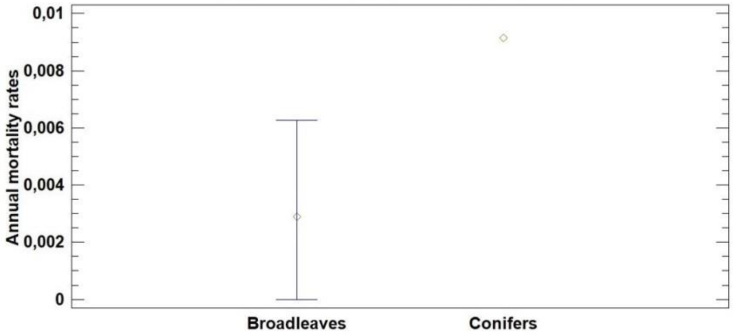

Figure S3. Median plot with 95% confidence intervals for the annual mortality rates of broadleaved and conifer tree species monitored in the territory of Serbia in the period of 2014–2018.

Table S3. Descriptive and nonparametric statistics for the annual mortality rates of trees monitored at different altitude ranges in the territory of Serbia in the period of 2014–2018.

Altitude range	Sample size	M	MAD	MIN	MAX	Average rank in KWt	Test statistic	*P*-value
0-500	67	0.000	0.000	0.000	0.032	72.463	3.4053	0.1822
501-800	28	0.000	0.000	0.000	0.058	59.323		
>801	39	0.000	0.000	0.000	0.028	64.795		

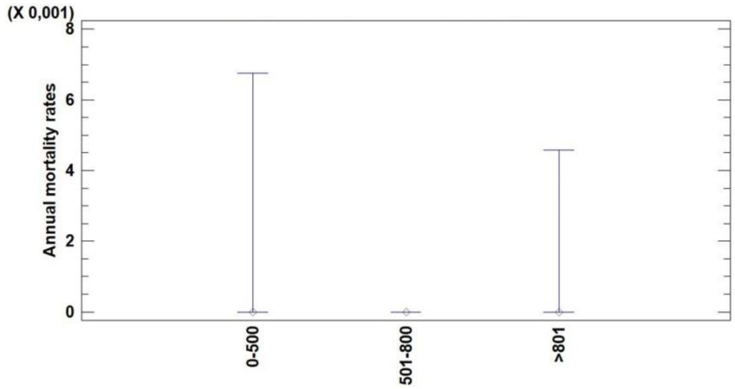

Figure S4. Median plot with 95% confidence intervals for the annual mortality rates of trees monitored at different altitude ranges in the territory of Serbia in the period of 2014–2018.

Table S4. Mean annual air temperatures (°C) in Serbia in the period from 2004 to 2018

Year	2004	2005	2006	2007	2008	2009	2010	2011	2012	2013	2014	2015	2016	2017	2018
**Mean**	10.6	10.0	10.7	**11.9**	**11.8**	11.5	11.1	10.9	**11.7**	**11.7**	**11.9**	11.8	11.4	11.5	**12.0**

Table S5. Mean annual air temperatures (°C) in the growing season in Serbia from 2004 to 2018

Year	2004	2005	2006	2007	2008	2009	2010	2011	2012	2013	2014	2015	2016	2017	2018
**Mean**	16.8	16.9	17.4	18.5	17.8	18.4	17.7	18.3	**19.4**	18.1	17.1	17.6	18.0	18.4	**19.1**

Table S6. Mean precipitation sum (mm) in Serbia in the period from 2004 to 2018

Year	2004	2005	2006	2007	2008	2009	2010	2011	2012	2013	2014	2015	2016	2017	2018
**Mean**	792.4	827.2	706.8	747.7	619.5	787.7	866.0	**472.2**	**614.4**	**664.6**	**1014.1**	675.2	856.8	624.4	789.9

Table S7. Mean precipitation sum (mm) in the growing season (April-September) in Serbia in the period from 2004 to 2018

Year	2004	2005	2006	2007	2008	2009	2010	2011	2012	2013	2014	2015	2016	2017	2018
**Mean**	413.0	511.5	422.8	328.8	345.7	334.6	476.5	**274.9**	**296.7**	**325.2**	**735.5**	321.3	447.4	327.6	423.7

Table S8. Comparative analysis of defoliation in the period 2004-2018 – Conifers

**Defoliation 2004-2018 – Conifers in %**															
	**2004**	**2005**	**2006**	**2007**	**2008**	**2009**	**2010**	**2011**	**2012**	**2013**	**2014**	**2015**	**2016**	**2017**	**2018**
**None** (0-10)	50,1	46,2	64,8	67,5	63,4	64,7	70,1	78,1	78,3	79	74,1	75,4	74,1	79,1	81,6
**Slight** (>10-25)	30	32,5	21,6	19,2	23,6	22,7	18	10,8	10,7	8	11,3	10,1	12,4	8,9	8,2
**Moderate** (>25-60)	19	20,1	11,8	9,7	10	10,2	9,2	7,8	7,7	8,6	8,6	8	8,1	8,9	6,2
**Severe** (>60<100)	0,9	1,2	1,2	2,1	3	2,4	2,7	3,3	3	4,1	5,4	4,4	3,6	3,1	4
**Dead** (100)	0	0	0,6	1,5	0	0	0	0	0,3	0,3	0,6	2,1	1,8	0	0
	100	100	100	100	100	100	100	100	100	100	100	100	100	100	100

Table S9. Comparative analysis of defoliation in the period 2004-2018 – Broadleaves

**Defoliation 2004-2018 – Broadleaves in %**															
	**2004**	**2005**	**2006**	**2007**	**2008**	**2009**	**2010**	**2011**	**2012**	**2013**	**2014**	**2015**	**2016**	**2017**	**2018**
**None** (0-10)	59,5	51,3	63,8	53,5	61	68,6	66,8	68,6	70,8	63,9	68,9	74,3	70,5	69,6	69,6
**Slight** (>10-25)	27	33	25,2	30,8	27,7	21,5	22,5	24,2	19	21,2	19	15,6	18,5	18,6	18,3
**Moderate** (>25-60)	12,6	15	10,6	14	9,9	8,6	8,8	6	7,6	10,1	8,6	6,4	7,9	10	9,5
**Severe** (>60<100)	0,9	0,7	0,3	1,5	1	0,7	1,0	0,6	1,8	3,9	2,7	3,2	2,3	1,7	2,5
**Dead** (100)	0	0	0,1	0,2	0,4	0,6	0,9	0,6	0,8	0,9	0,8	0,5	0,8	0,1	0,1
	100	100	100	100	100	100	100	100	100	100	100	100	100	100	100

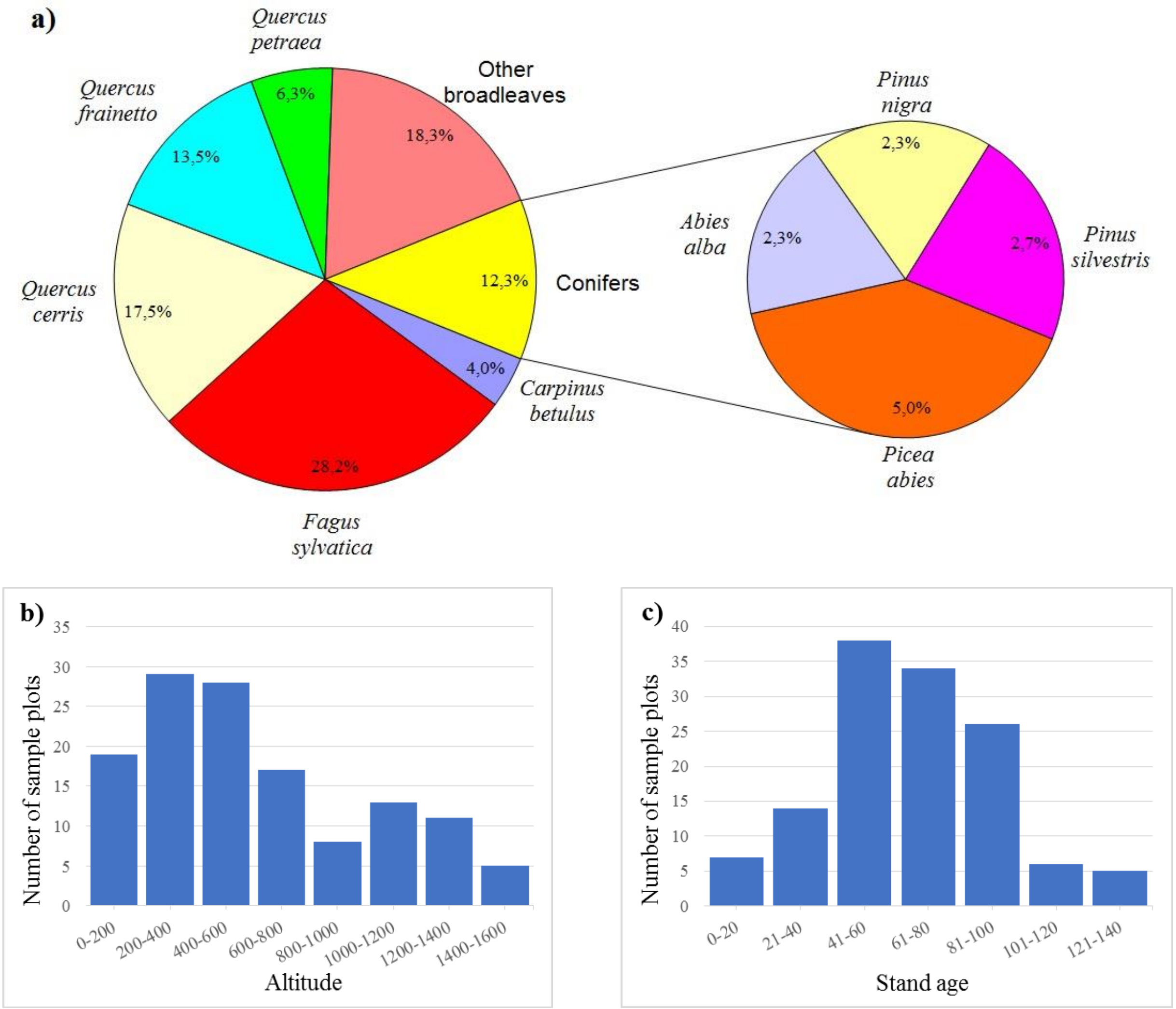

Figure S5. Distribution of 130 Sample plots by **a)** Tree species **b)** Altitude **c)** Stand age
